# Classification of weakly carcinogenic human papillomavirus types: addressing the limits of epidemiology at the borderline

**DOI:** 10.1186/1750-9378-4-8

**Published:** 2009-06-01

**Authors:** Mark Schiffman, Gary Clifford, Franco M Buonaguro

**Affiliations:** 1 Division of Cancer Epidemiology and Genetics, National Cancer Institute, US National Institutes of Health, Bethesda, Maryland, 20892, USA; 2 International Agency for Research on Cancer, F-69372 Lyon cedex 08, France; 3 Unit of Viral Oncogenesis and Immunotherapy, Istituto Nazionale Tumori, "Fond G. Pascale", Naples, Italy

## Abstract

Virtually all cases of cervical cancer are caused by persistent infections with a restricted set of human papillomaviruses (HPV). Some HPV types, like HPV16 and HPV18, are clear and powerful carcinogens. However, the categorization of the most weakly carcinogenic HPV types is extremely challenging. The decisions are important for screening test and vaccine development. This article describes for open discussion an approach recently taken by a World Health Organization International Agency for Research on Cancer (IARC) Monographs Working Group to re-assess the carcinogenicity of different HPV types.

## Background and rationale

As a group, human papillomaviruses (HPV) are proven human carcinogens. But there are >> 100 HPV genotypes and only a small fraction have any known carcinogenic potential [1]. Therefore, moving from broad acceptance of the carcinogenicity of persistent HPV infection to specific conclusions about individual genotypes requires consideration of each type as an individual agent. Such a type-by-type evaluation proves to be very difficult, and stretches epidemiology to its limits because of issues of confounding and misclassification detailed below. However, as described in the accompanying article by Castle [2], it is important for the development of screening tests and vaccines to judge each HPV type separately; thus, it is worth considering how well one actually can decide whether a given HPV type is carcinogenic or not. This article describes an approach taken by a Working Group of the World Health Organization International Agency for Research on Cancer (IARC), when asked to re-assess for official purposes which HPV types should be grouped as carcinogens [3]. The process is described here to promote needed debate on what an improved approach might be.

IARC formally considers the carcinogenicity of exposures to humans. Whereas human carcinogenicity might best be considered for some agents like HPV as a continuum of probabilities without a clear breaking point, IARC classifies carcinogens categorically as carcinogenic (Group 1), probably carcinogenic (Group 2a), possibly carcinogenic (Group 2b), not classifiable (Group 3), or probably not carcinogenic (Group 4). There has been very little experimental work on the carcinogenicity of HPV types except for HPV16 and HPV18; thus, epidemiologic evidence has been unusually important. Epidemiologic evidence for the carcinogenicity of HPV was originally presented in IARC Monograph Volume 64 [4], and was extensively updated in IARC Monograph Volume 90 based on data available as of February 2005 [5]. In February 2009, Volume 100b updated the data once again and it is this latest update addressed here. For the purposes of IARC, epidemiologic evidence is categorized as sufficient, limited, inadequate or suggesting lack of carcinogenicity. The epidemiologic data are combined with experimental evidence (in this case lacking for most HPV genotypes) to arrive at the final Groups 1–4.

HPV carcinogenicity has been established most convincingly for cervical cancer, and this discussion will be limited to cervical carcinogenicity. To date, no HPV type has been proven to be carcinogenic only at sites other than the cervix.

## HPV evolution as the guiding principle of HPV behaviour (the high-risk clade)

**Figure 1:**
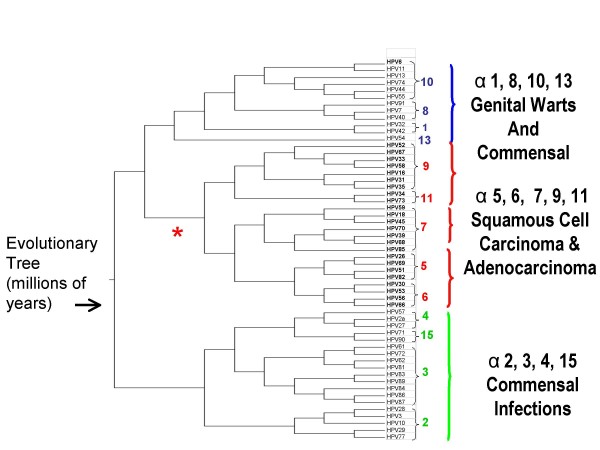
**Phylogenetic analysis of anogenital HPV types **[6]. Branches determined by 100 bootstrap estimations using each of the methods in the following order: Bayesian credibility value, parsimony bootstrap percentage based on nucleotide alignment, and parsimony bootstrap percentage based on amino acid alignment. All definitely, probably, and possibly carcinogenic HPV types belong to one phylogenetic clade of the alpha genus.

HPV behaviour at the cervix is strongly correlated with phylogenetic (i.e. evolutionary) categories [6]. All HPV genotypes that are known to be cervical carcinogens belong to the alpha genus in an evolutionary branching or clade containing a few genetically related species (Figure 1). Epidemiologic data do not support cervical carcinogenicity for other species in the alpha genus or for other genera. To save considerable space presenting null evidence, this section will not include data related to HPV species alpha-1, -2, -3, -4, -8, -10 (other than HPV 6), -13, or 14/15. These species contain HPV types that cause skin or genital warts, minor cytologic atypia, and often no apparent disease.

In Monograph 64 in 1995, HPV 16 (alpha-9) and HPV 18 (alpha-7) were classified as cervical carcinogens. HPV 31 and HPV 33 in alpha-9 were categorized as probably carcinogenic [4]. In 2005, the group of cervical carcinogens (Group 1) was expanded to include the following 13 types: alpha-5 genotype HPV 51; alpha-6 genotypes HPV 56 and HPV 66; alpha-7 genotypes HPV 18, HPV 39, HPV 45, and HPV 59; and alpha-9 genotypes HPV 16, HPV 31, HPV 33, HPV 35, HPV 52, and HPV 58 [5].

In the four years between Monograph 90 in 2005 and the recent update, new evidence further supported that HPV types in the high-risk clade of the alpha genus HPV type cause virtually all cases of cervical cancer worldwide [7,8]. In case-control studies, the odds ratios associating cervical cancer and its immediate precursor, CIN3, with HPV DNA positivity for these types in aggregate has consistently exceeded 50. It is persistent infections that are associated with extremely high absolute risk of CIN3 and cancer. In cohort studies, women who test negative to this group of HPV types are at extremely low subsequent risk of CIN3, cancer, and cancer death for more than 10 years [9-11].

Because persistent infection with a restricted group of HPV types is a nearly necessary cause of cervical cancer, a reconsideration of HPV and cervical carcinogenicity based on the new data must decide whether any additional types within the high-risk clade are also carcinogenic and whether any types in that clade that were previously categorized as carcinogenic should be downgraded. The types in the high-risk clade are listed in Table 1.

**Table 1:** HPV Types that Were Considered in Monograph 100B

Alpha Species	Types Categorized as Definite Carcinogens in Monograph 90	Other Types in Species
5	51	26 69 82

6	56 66	30 53

7	18 45 39 59	68 70 85 97

9	16 31 33 35 52 58	67

11		34 73

## Conceptual issues in deciding which of the types in the high-risk clade are carcinogenic

From a virologic perspective, the definitive proof of carcinogenicity of an HPV type is finding transcriptionally active HPV in a tumour. HPV is not a "hit and run" carcinogen, and transcriptional activity is needed for maintenance of the cancer phenotype. In cervical cancer cell lines, blocking transcriptional activity by antisense RNA leads to apoptosis. This level of evidence is simply lacking for virtually all HPV types. The vast body of evidence relates simply to finding HPV DNA at the same time of cervical neoplasia.

However, relying on testing of scrapes or biopsies, by DNA testing, leads to difficulties. The alpha HPV types share a common route of transmission and multiple infections are present in a large minority of women, although they might not be transmitted from the same partner or at the same time. Given the existence of some very powerfully carcinogenic types, notably HPV 16 and HPV 18, determining which weaker and/or less common types are also carcinogenic becomes (for the epidemiologist) an issue of confounding. None of the traditional approaches to control of confounding are entirely successful. Because HPV 16 causes approximately 50% of cases of cervical cancer, logistic regression and similar approaches will parsimoniously attribute cases associated with both HPV16 and a less important type to HPV16. HPV 18 is the second most important cervical carcinogen, responsible for approximately 15–20% of cervical cancer of all histologic types combined (and a higher fraction of adenocarcinomas). If a type occurs with either HPV 16 or HPV 18, its association with cervical cancer might be confounded by either of these powerful carcinogens. For types causing only a very small fraction of cervical cancer, confounding by any of the more important types is possible.

Dealing with confounding by exclusion, i.e. examining the possibility of carcinogenicity of a more minor type among cancer specimens that do not contain a more important type, becomes a problem of misclassification. This main epidemiologic criterion used for classification of an HPV type as a carcinogen, finding the HPV genotype as a single infection in a cervical scrape or biopsy specimen in a woman with cancer, might sometimes be too lax and prone to error. Colposcopic biopsies and cytology specimens can be misdirected and fail to obtain the critical cells, while contamination of scrapes and biopsies from lower-grade lesions that often surround cancers can detect types other than the causal one. Studies relying on testing of microdissected cervical malignancies will address these issues, but large-scale highly accurate data are not yet available.

Difficulty with control selection adds another level of complexity in assessing carcinogenicity. Cervical cancer typically follows age infection by decades. HPV transmitted at young ages usually become undetectable by DNA or RNA assays and no sensitive serologic assay exists to measure HPV exposure. Consequently, odds ratios based on a comparison of HPV prevalence at the time of case diagnosis to age-matched HPV point prevalence in controls do not estimate true relative risks.

There is not much type-specific prospective data on the carcinogenicity of individual HPV genotypes. The available studies have categorically shown the unique carcinogenicity of HPV 16 and, to a lesser extent, HPV 18 [9,12]. Khan et al. observed a risk for the remaining women positive by hc2 after excluding those positive for HPV 16 or HPV 18 (including an unknown mix of the types 31, 33, 35, 39, 45, 51, 52, 56, 58, 59 and 68) of only 3.0% (1.9–4.2) compared with 0.8% (0.6–1.1) among women who were HPV negative at baseline. Thus, there is not strong and convincing long-term prospective evidence for individual HPV types other than HPV16 and HPV18.

Finally, the accuracy of detection of HPV genotypes differs between the major PCR-based systems used to generate most of the data [13]. Each of the systems shows differential sensitivity, and some have exhibited cross-reactivity of detection. These detection issues are not critical for evaluating the most important HPV types, but make it difficult to clarify the role of the most weakly carcinogenic and least common ones.

With these caveats, the cervical carcinogenicity of the HPV types listed above varies in strength in a continuum without clear breakpoint, from extremely strong (i.e. HPV 16 and, to a lesser degree, HPV 18) to weak, but still probably carcinogenic in rare instances (e.g. HPV 68, see below). Evaluators taking one extreme position could claim that there is reasonable evidence for carcinogenicity of virtually all the types in the species listed above, extending further the list established in Monograph 90. Strict interpreters of causal criteria could argue for a return to a much more limited list. But based on current evidence, no cut-point between sufficient, limited, and inadequate epidemiologic evidence is entirely defensible.

## Approach taken by the IARC working group

### Data Sources

The IARC Working Group chose the following pragmatic approach to creating an imperfect cut-point between sufficient, limited, and inadequate epidemiologic evidence for cervical carcinogenicity: Only types in the high-risk clade were considered because data seemed grossly inadequate for all others. The most recent accumulated data on type-specific HPV prevalence in cervical cancers were drawn from a very large single project [7] and from meta-analyses performed by IARC ([8] updated by G. Clifford). Excluded from consideration were high-grade precancerous lesions (CIN3 and the more equivocal CIN2 which occur in approximately 1% of screened women), which often used as ethical surrogate endpoints in prospective studies and clinical trials; there are now sufficient data for invasive cancers and it appears that HPV types have different potential to progress from CIN2/3 to invasive cervical cancer [14]. The background frequency of cervical HPV infection in the general female population was estimated from a large meta-analysis of HPV genotypes found in women with normal cytology [15], as shown in Table 2. Although women included in meta-analyses of cervical cancer and normal cytology differed by age, region and HPV testing protocols, it was considered the best method to identify a reasonable threshold of confounding and misclassification for each type. Ancillary analyses examining the issue of most important types by region were also scrutinised.

**Table 2:** Meta-analyses of type-specific HPV DNA prevalence in invasive cervical cancer [15] and women with normal cytology [14,17]

	**Invasive cervical cancer**			**Normal**		
	**N tested**	**% pos**	**95% CI**	**N tested**	**% pos**	**95% CI**
**HPV16**	14595	54.4	53.6–55.2	76385	2.6	2.5–2.8
**HPV18**	14387	15.9	15.3–16.5	76385	0.9	0.8–1.0
**HPV33**	13827	4.3	4.0–4.6	74141	0.5	0.4–0.5
**HPV45**	9843	3.7	3.3–4.1	65806	0.4	0.4–0.4
**HPV31**	11960	3.5	3.2–3.9	74076	0.6	0.6–0.7
**HPV58**	10157	3.3	2.9–3.6	72877	0.9	0.8–1.0
**HPV52**	9509	2.5	2.2–2.8	69030	0.9	0.8–1.0
**HPV35**	9507	1.7	1.5–2.0	74084	0.4	0.3–0.4
						
**HPV59**	6972	1.0	0.8–1.3	64901	0.3	0.2–0.3
**HPV51**	7339	0.7	0.5–0.9	67139	0.6	0.6–0.7
**HPV56**	7427	0.7	0.5–0.9	68121	0.5	0.5–0.6
**HPV39**	7078	0.6	0.5–0.9	64521	0.4	0.3–0.4
						
**HPV68**	6723	0.5	0.3–0.7	63210	0.3	0.2–0.3
**HPV73**	5837	0.5	0.3–0.7	44063	0.1	0.1–0.1
						
**HPV66**	6664	0.3	0.2–0.5	59774	0.4	0.3–0.4
**HPV70**	5159	0.2	0.1–0.4	35014	0.3	0.3–0.3
**HPV82**	5352	0.1	0.1–0.3	42536	0.1	0.0–0.1
						
**HPV6**	9911	0.5	0.4–0.7	58370	0.3	0.2–0.3
						
**HPV53**	not reported			44,058	0.4	0.4–0.4
**HPV26**	not reported			44,098	0.0	0.0–0.1
						
**HPV85**	not studied in ICC meta-analysis			9,622	0.1	0.1 – 0.1
**HPV67**	not studied in ICC meta-analysis			18,041	0.0	0.0 – 0.0
**HPV34**	not studied in ICC meta-analysis			42,588	0.0	0.0 – 0.1
**HPV30**	not studied in ICC meta-analysis			8,773	0.0	0.0 – 0.1
**HPV97**	not studied in ICC meta-analysis			not studied in normal cytology meta-analysis		

### Uniqueness of HPV16 and HPV18

Comparing the prevalences in women with normal cytology to the prevalences for cancers compiled by Smith et al. (2007) (Table 2), obvious "case-control" differences can be seen. The most clearly carcinogenic genotypes, HPV 16 and HPV 18 in particular, are more common among cancers and cytologically normal women (and even low-grade lesions [16]). HPV 18 is especially common in adenocarcinomas [17], as are other members of the alpha 7 clade of which HPV18 is a member. The large amount of data regarding HPV 16 and HPV 18 was thought to provide ample epidemiologic evidence of carcinogenicity (leading to an overall classification of Group 1).

### The 8 most important carcinogenic HPV types

Including HPV 16 and HPV 18, eight HPV types (alpha-7 types HPV 18 and 45, alpha-9 types 16, HPV 31, 33, 35, 52 and 58) are the most common types found in cancers in both the IARC meta-analysis [8] and the ICO study [7], in all regions of the world providing data. Though very often found in non-invasive lesions, these types are all much more common in cancer case specimens than in controls, providing sufficient epidemiologic evidence of carcinogenicity (Group 1).

### The borderline carcinogens: using HPV 6 as an estimator of residual confounding

To move beyond the most evidently carcinogenic eight HPV types, the Working Group chose an estimator of the percentage of cancers that might contain HPV DNA by accumulated and unknown measurement errors alone. The group made use of HPV 6 for this estimation. Specifically, HPV 6, the important and common cause of benign condyloma acuminata (external genital warts) was considered to be a low-risk type, not classified as a cervical carcinogen, which only uncommonly is detected in cervical cancer specimens. [Of note, it remains possible that HPV 6, and other "low risk types" can cause cancer in extremely rare virus-host circumstances.] When detected, even without detection of a more likely causal type, the Working Group judged that misclassification of some kind was a more likely explanation than causality. As given in Table 3, the best IARC estimate of detection of HPV 6 in cervical cancers, not necessarily as a single infection, was judged to be 0.45%, 95% CI 0.35 – 0.56, based on 14,912 cases of cancer ([8] updated by G. Clifford for the Working Group). The Working Group pragmatically made the following rule: An individual HPV type in the high-risk alpha clade (i.e. one with an elevated prior probability of being carcinogenic due to analogy to closely related viral types in the same or closely-related species) was considered to have sufficient epidemiologic evidence of carcinogenicity if its prevalence in cancers was 1) significantly greater than that of HPV 6, and 2) significantly enriched in comparison to the background estimate for the general population, i.e. women with normal cytology.

**Table 3:** Meta-analysis of type-specific HPV DNA prevalence in invasive cervical cancer [15], updated with 63 newly published studies by Clifford (IARC)

	**N tested**	**N pos**	**% pos**	**95% CI**
**hpv39**	13370	172	1.29	1.10 – 1.48
**hpv59**	13471	172	1.28	1.09 – 1.47
**hpv51**	13057	151	1.16	0.97 – 1.34
**hpv56**	13247	103	0.78	0.63 – 0.93
				
**hpv68**	11982	73	0.61	0.47 – 0.75
**hpv73**	9939	48	0.48	0.35 – 0.62
				
**hpv6**	14912	68	0.45	0.35 – 0.56
				
**hpv53**	8140	34	0.42	0.28 – 0.56
**hpv66**	12118	47	0.39	0.28 – 0.50
**hpv70**	10503	35	0.33	0.22 – 0.44
**hpv82**	9265	25	0.27	0.16 – 0.38
**hpv26**	6111	8	0.13	0.04 – 0.22

HPV types 30, 34, 67, 85 and 97 were not studied in the meta-analysis dataset.

By this logic, four more types were categorized as having sufficient epidemiologic evidence leading to their classification as definite (Group 1) carcinogens: alpha-5 type 51 (1.16% of cervical cancer), alpha-6 type 56 (0.78%), and alpha-7 HPV types 39 (1.29%) and 59 (1.28%).

### Classification of other types in the high-risk clade

The remaining types in the high-risk alpha clade were considered, as a group, to have limited evidence to support carcinogenicity. If phylogeny can be taken to predict behaviour, it is possible that most of these types can very rarely cause cancer. Indeed, many of the types have been detected, albeit uncommonly (not significantly greater than HPV 6), in cancers. There are not enough data, even after testing of many thousands of specimens, to be sure which ones are carcinogenic or not. Furthermore, some of these types, namely 30, 34, 67, 85 and 97 have not yet been studied in the IARC cervical cancer meta-analysis approach. Nevertheless, within this group, there are two types, alpha-7 type HPV 68 and alpha-11 type HPV 73, for which the data are slightly stronger than for the others despite methodologic challenges. One of the major PCR-based testing methods (SPF10) cannot distinguish these two types because their amplicons using those primers are identical [18]. Neither of these two types is optimally detected by MY09-MY11 dot blot [19]. Nonetheless, the data supporting the carcinogenicity of HPV 68 and HPV 73 are very suggestive although not sufficient. Ultimately, the existence of a cell line whose immortalization is sustained by a subtype of HPV68 (ME180), led the Working Group to classify HPV 68 as a probable carcinogen (Group 2A). The rest were called possible (Group 2B).

### Re-classification of HPV 66

Overall, the Working Group approach led to the re-classification from Monograph 90 of HPV 66 to possible (Group 2B), although the epidemiologic evidence of carcinogenicity was previously judged sufficient (Group 1). In the assembly of much more testing data from cancer cases, HPV 66 has been found so rarely that its percentage of detection is less than the relative percentage of detection among the general population. In the Working Group review of each individual article, HPV 66 was found as a single infection in cancers with extreme rarity, well below the threshold of possible confounding and misclassification.

## Summary

There is no perfect way to categorize a continuum of carcinogenic potential. The Working Group arrived at the following conclusions, with a healthy scepticism concerning the process:

a) Persistent HPV 16 infection is a uniquely powerful human carcinogen.

b) HPV 18 is also important, particularly for adenocarcinoma.

c) Six additional types in alpha-7 (HPV 45) and alpha-9 (HPV 31, HPV 33, HPV 35, HPV 52, HPV 58) comprise the remainder of the eight types that are the most important globally, with some regional variation in the etiologic fractions of cancers due to each type.

d) There are small and less certain, incremental etiologic contributions of another group of carcinogenic types from alpha-5 (HPV 51), alpha-6 (HPV 56), and alpha-7 (HPV 39 and HPV 59). Each causes a few percent at most of cervical cancer cases worldwide. There has not been any large-scale study of regional variability for these uncommon types

e) There is an unresolved dividing line between the HPV types with the weakest evidence judged to be sufficient and those judged to have highly suggestive yet limited data (e.g. alpha-7 HPV 68 is categorized as probably carcinogenic due to experimental evidence while alpha-11 HPV 73 is possible).

f) The expanded data for HPV66 were re-evaluated and the evidence was judged to be very limited now that more cases have been studied showing that it is very rarely found in cancers despite being relatively common in the newly collated data on women with normal cytology. HPV 53, also in alpha species 6, shows the same pattern of relative common population prevalence with extremely rare cases of occurrence alone in cancer. The Working Group noted that for these types in particular, there could be harm to public health if the types are viewed with excessive concern; including these types as carcinogenic in screening assays would decrease the specificity and positive predictive value of the assays with virtually no gain in sensitivity and negative predictive value [20].

g) There are several types within the high-risk clade that have extremely sparse or no evidence of carcinogenicity. For some types there are anecdotal but very interesting cases that merit pursuit of additional carcinogenic types. There have been potentially underappreciated reports of alpha-9 type HPV 70 found as single infections in cancer, but the supportive data are sparse. There are only a few reports of HPV 67 in cancer [21-24], which is intriguing because this is the only known type in the alpha-9 species that is not categorized as carcinogenic. For a few types in the high-risk clade, no reports of invasive cancers with single-type infections were found, but isolated reports might exist.

h) The possible role of immunosuppression in HPV carcinogenicity was not emphasized in the Working Group discussions. To cause cancer, an HPV infection must persist and it is possible that some HPV types are only weak carcinogens because they persist poorly. For example, the carcinogenicity of alpha-5 type HPV 26 has been supported by a recent report of multiple peri-ungual cancers in an immunosuppressed individual, containing high viral loads and active transcription of HPV 26 alone [25]. HPV 26 is an uncommon type; perhaps the immunosuppression in this individual was a significant contributor to carcinogenesis. As one avenue of research, there should be more studies of whether HPV types in invasive cancers in HIV-infected individuals differ in type from the types found in immunocompetent individuals [25].

## Conclusion

When epidemiology serves as the science informing public health policy, its limitations must be acknowledged. Weak causal associations with one HPV type are extremely hard to prove in the presence of powerful confounding by strong carcinogens like HPV16. A coming generation of intensive molecular studies of microdissected cancers, with examination of transcriptional activity, may clarify the borderline between carcinogenic and non-carcinogenic HPV types. For public health purposes, the main agents that merit consideration in screening tests and vaccines have already been identified.

## References

[B1] Schiffman M, Castle PE, Jeronimo J, Rodriguez AC, Wacholder S (2007). Human papillomavirus and cervical cancer. Lancet.

[B2] Castle P (2009). The Evolving Definition of Carcinogenic Human Papillomavirus Infectious. Agents and Cancer.

[B3] Bouvard V, Baan R, Straif K, Grosse Y, Secretan B, El Ghissassi F, Benbrahim-Tallaa L, Guha N, Freeman C, Galichet L, Cogliano V, WHO International Agency for Research on Cancer Monograph Working Group (2009). A review of human carcinogens – Part B: biological agents. Lancet Oncol.

[B4] IARC Monographs Human Papillomaviruses. IARC Monographs on the Evaluation of Carcinogenic Risks to Human.

[B5] Cogliano V, Baan R, Straif K, Grosse Y, Secretan B, El Ghissassi F, WHO International Agency for Research on Cancer (2005). Carcinogenicity of human papillomaviruses. Lancet Oncol.

[B6] Schiffman M, Herrero R, Desalle R, Hildesheim A, Wacholder S, Rodriguez AC, Bratti MC, Sherman ME, Morales J, Guillen D, Alfaro M, Hutchinson M, Wright TC, Solomon D, Chen Z, Schussler J, Castle PE, Burk RD (2005). The carcinogenicity of human papillomavirus types reflects viral evolution. Virology.

[B7] Bosch FX, Burchell AN, Schiffman M, Giuliano AR, de Sanjose S, Bruni L, Tortolero-Luna G, Kjaer SK, Muñoz N (2008). Epidemiology and natural history of human papillomavirus infections and type-specific implications in cervical neoplasia. Vaccine.

[B8] Smith JS, Lindsay L, Hoots B, Keys J, Franceschi S, Winer R, Clifford GM (2007). Human papillomavirus type distribution in invasive cervical cancer and high-grade cervical lesions: a meta-analysis update. Int J Cancer.

[B9] Khan MJ, Castle PE, Lorincz AT, Wacholder S, Sherman M, Scott DR, Rush BB, Glass AG, Schiffman M (2005). The elevated 10-year risk of cervical precancer and cancer in women with human papillomavirus (HPV) type 16, or 18, and the possible utility of type-specific HPV testing in clinical practice. J Natl Cancer Inst.

[B10] Sankaranarayanan R, Nene BM, Shastri SS, Jayant K, Muwonge R, Budukh AM, Hingmire S, Malvi SG, Thorat R, Kothari A, Chinoy R, Kelkar R, Kane S, Desai S, Keskar VR, Rajeshwarkar R, Panse N, Dinshaw KA (2009). HPV screening for cervical cancer in rural India. N Engl J Med.

[B11] Schiffman M, Wacholder S (2009). From India to the world – a better way to prevent cervical cancer. N Engl J Med.

[B12] Wheeler CM, Hunt WC, Schiffman M, Castle PE, Atypical Squamous Cells of Undetermined Significance/Low-Grade Squamous Intraepithelial Lesions Triage Study Group (2006). Human papillomavirus genotypes and the cumulative 2-year risk of cervical precancer. J Infect Dis.

[B13] Gravitt PE, Coutlée F, Iftner T, Sellors JW, Quint WG, Wheeler CM (2008). New technologies in cervical cancer screening. Vaccine.

[B14] Clifford GM, Smith JS, Aguado T, Franceschi S (2003). Comparison of HPV type distribution in high-grade cervical lesions and cervical cancer: a meta-analysis. Br J Cancer.

[B15] de Sanjosé S, Diaz M, Castellsagué X, Clifford G, Bruni L, Muñoz N, Bosch FX (2007). Worldwide prevalence and genotype distribution of cervical human papillomavirus DNA in women with normal cytology: a meta-analysis. Lancet Infect Dis.

[B16] Clifford GM, Rana RK, Franceschi S, Smith JS, Gough G, Pimenta JM (2005). Human papillomavirus genotype distribution in low-grade cervical lesions: comparison by geographic region and with cervical cancer. Cancer Epidemiol Biomarkers Prev.

[B17] Clifford G, Franceschi S (2008). Members of the human papillomavirus type 18, family (alpha-7 species) share a common association with adenocarcinoma of the cervix. Int J Cancer.

[B18] Molijn A, Kleter B, Quint W, van Doorn LJ (2005). Molecular diagnosis of human papillomavirus (HPV) infections. J Clin Virol.

[B19] Handisurya A, Rieger A, Bankier A, Koller A, Salat A, Stingl G, Kirnbauer R (2007). Human papillomavirus type 26, infection causing multiple invasive squamous cell carcinomas of the fingernails in an AIDS patient under highly active antiretroviral therapy. Br J Dermatol.

[B20] Schiffman M, Khan MJ, Solomon D, Herrero R, Wacholder S, Hildesheim A, Rodriguez AC, Bratti MC, Wheeler CM, Burk RD, PEG Group; ALTS Group (2005). A study of the impact of adding HPV types to cervical cancer screening and triage tests. J Natl Cancer Inst.

[B21] Gudleviciene Z, Didziapetriene J, Ramael M, Uleckiene S, Valuckas KP (2006). Human papillomavirus and p53 polymorphism in Lithuanian cervical cancer patients. Gynecol Oncol.

[B22] Wentzensen N, Schiffman M, Dunn ST, Zuna RE, Walker J, Allen RA, Zhang R, Sherman ME, Wacholder S, Jeronimo J, Gold MA, Wang SS (2009). Grading the severity of cervical neoplasia based on combined histopathology, cytopathology, and HPV genotype distribution among 1,700 women referred to colposcopy in Oklahoma. Int J Cancer.

[B23] Gargiulo F, De Francesco MA, Schreiber C, Ciravolo G, Salinaro F, Valloncini B, Manca N (2007). Prevalence and distribution of single and multiple HPV infections in cytologically abnormal cervical samples from Italian women. Virus Res.

[B24] Andersson S, Mints M, Sällström J, Wilander E (2005). The relative distribution of oncogenic types of human papillomavirus in benign, pre-malignant and malignant cervical biopsies. A study with human papillomavirus deoxyribonucleic acid sequence analysis. Cancer Detect Prev.

[B25] De Vuyst H, Gichangi P, Estambale B, Njuguna E, Franceschi S, Temmerman M (2008). Human papillomavirus types in women with invasive cervical carcinoma by HIV status in Kenya. Int J Cancer.

